# Criticism of Dental Colleges

**Published:** 1891-10

**Authors:** 


### Editorial.

### CRITICISM OF DENTAL COLLEGES.

Legitimate criticism is always valuable, whether applied to persons or institutions, and if this is made in the right spirit it becomes a powerful stimulus to progress. The use and not the abuse of this power has had much to do with forcing the dental profession to make advanced steps; indeed it is questionable whether in educational matters dentistry would to-day be not where it was twenty years ago had it not been subjected to this both at home and abroad.

The natural tendency of the human mind is towards conservatism,—a willingness to let things rest quietly in present grooves, and deprecating any disturbing element. The active colleges of this country were exactly in this condition less than a decade ago, and but for this outside force would probably be in the same pitiable state to-day. Their inability to throw off the accumulation of years of erroneous practice was so clearly apparent that the organization known as the Association of Dental Faculties became an absolute necessity. Through the combined operation of internal and external forces, dental education has been brought to a position worthy the respect of all classes and of all professions.

Criticism, being disarmed on the old lines, has begun an open attack on the internal management of these schools, and a sample of this was given our readers in the September number of the Journal, entitled "The Use and Abuse of Dental Charity," by Richard Grady, M.D., D.D.S.

We would not deem it our duty to make any observations upon this paper had it given intrinsic evidence that the subject-matter discussed had been obtained by personal observation and experience, and not secondarily, from loose statements of individuals or letters from deans of colleges.

The criticism of the writer in general has force, for there can be no question but that abuses have arisen in many colleges, perhaps to a limited extent in all, and need careful attention.

The paper claims to be a carefully-prepared document, based on answers received from various persons in authority. Accepting this somewhat uncertain method of acquiring information, the writer proceeds to build up a theory which seems to us as weak as the foundation upon which it is laid.

It was early found by the fathers that the only possible way of teaching dentistry was to abandon the old methods familiar in medical schools, and mainly confined to didactic lectures, and, while not excluding these, adding thereto practical work on the living subject. The years of experience that have elapsed since then have confirmed the wisdom of this view, and the student of to-day, with natural ability, on graduating will compare favorably with older practitioners in oral manipulations. This cannot be said of any other profession, and is unquestionably due to the methods adopted.

We do not understand the essayist to dispute this well-understood fact, but he persists in regarding the present plan as an "abuse of dental charity," and that its tendency is to pauperize the people.

The charge that colleges are making money is dwelt upon at some length, as though this was something no college had a right to do. To make money means with him "an abuse of dental charity." Yet it has never been shown, or acknowledged by the colleges, that they were charitable organizations in any sense of the word. The object of their existence is to educate young men in dentistry, and if, in so doing, the income exceeds expenses, it is not a matter that need concern either the profession or the general public.

The point to be considered is not whether money is or is not made, but whether this mode of training be the correct one. The college faculties recognize the fact that no other available mode exists, and to abandon it now would mean the destruction of dental education. The old method of preceptor-training was, and is, an admitted failure, for the reason that the student rarely could get beyond the mechanical laboratory, oral operations being prohibited. With present methods theory goes hand-in-hand with practice, and so thoroughly interwoven into the mental nature of the student as to be an ever-continuing active force.

While colleges are not working on altruistic lines, yet the importance of the labor performed by them for the good of the people is incalculable, and outweighs any injury the practice adopted may have been to individuals. So thorough has been this training in our large cities, that the masses now fully recognize the value of dental work, and are willing to pay for it as they never have been before, and in proportion to this knowledge has the health and comfort of large bodies of people been increased.

The essayist seems to recognize only one side of the question, and that the selfish one, its effect being to "lower the dignity or emoluments of private practice," "warring upon the livelihood of their professional brethren," and, consequently, it is an injury to the profession in all cities afflicted with colleges. It lowers the standard of remuneration. It invites persons who can pay to have their work done at rates below a living basis. It lowers the profession in public estimation and debases all concerned. This in substance constitutes the arraignment.

The remedy for this, as stated, demonstrates very clearly that his experience has not been an extended one in the management of dental colleges. His panacea for the ills enumerated is to place a sign at the entrance door, "For the poor only." A very limited knowledge of human nature would settle that question. The idea of "poor" in the mind of the essayist seems to be those under the care of "charity organizations," or perhaps the residents of those wretched quarters common to all cities,—the last resting-places of vagrant humanity in life. Neither of these classes are proper subjects for the dental infirmary. The families of the laboring poor is the class next above; but these cannot spare the time, however much they may desire it, to visit, day after day, the operating-room for careful attention.

The class of really poor but very respectable working-girls in factories and stores is the one which fills our college infirmaries. It is a mistake to suppose these latter are patronized by the wealthy. It is true that occasionally a person actuated by miserly motives, or under the idea that they will secure better operations, will visit these institutions; but an experience of many years, from demonstrator to principal, in college work, leads the writer of this to the conclusion that these cases are exceptional, and that in reality the labor is done for the poor, and that at reasonable rates, and in this sense may be strictly within the lines of charity.

Experience has demonstrated that no rule will answer that attempts to discriminate between the amount of wealth of individuals. To ask a person, "Are you able to pay?" means an insult to be resented. To judge by the clothing opens the door to failure. To charge fees equal to those received in private practice means destruction to this mode of training. In a word, no plan has been suggested that will help to solve this problem, and any attempt to force matters by protective associations must prove a failure.

Education must advance in the progress of evolution of methods. Change means injury in certain directions. The forces that underlie all advances are destructive in their character for a time, and then comes the period of equilibrium and the interval of compensations. The advent of machinery caused ruin and sorrow to thousands, but in the advancing years brought comfort and measurable competence to an indefinite number. The injury that the essayist complains of may be a real one to the few, but that it is a blessing to thousands outside of cities must be clear to the intelligent observer. Colleges have filled every town and hamlet with an educated and trained class of men, and at the same time driven out the ignorant, untrained, travelling tooth-extractor.

In conclusion, we must regard Dr. Grady's paper as valuable, in that it will stir thought in this direction; but that its tenor is simply iconoclastic must be conceded. It gives no evidence of ability to build better than the experience of a half-century has demonstrated as the only possible way to educate the dental fraternity of the present epoch.

